# Supplementary material for: Spatial transcriptomics of a parasitic flatworm provides a molecular map of drug targets and drug resistance genes
Source: Nat Commun. 2024 Oct 16;15:8918. doi: 10.1038/s41467-024-53215-3 (PMC11484910; doi:10.1038/s41467-024-53215-3)
Supplement: Supplementary file 1 — Supplementary Information [file 41467_2024_53215_MOESM1_ESM.pdf]

Supplementary Information

Spatial transcriptomics of a parasitic flatworm provides a molecular map of drug targets and drug-resistance genes

Svenja Gramberg[1], Oliver Puckelwaldt[1], Tobias Schmitt[1], Zhigang Lu[2], Simone Haeberlein[1*]

[1]Institute of Parasitology, Justus Liebig University Giessen, Giessen, Germany
[2]Institute of Food Science and Biotechnology, University of Hohenheim, Stuttgart, Germany
*Corresponding author: Simone.Haeberlein@vetmed.uni-giessen.de

Table of contents

Supplementary Figures 1-10

Supplementary Figure 1. 10x Visium preparation and tissue optimization

Supplementary Figure 2. Tissue composition and QC metrics of all four specimens in the liver fluke spatial transcriptomics dataset

Supplementary Figure 3. Spatial expression of tegument and muscle markers

Supplementary Figure 4. Spatial expression of Giotto metagenes 9-15

Supplementary Figure 5. The parasite's egg production apparatus: from vitellarium to uterus

Supplementary Figure 6. The liver fluke's Mehlis' gland is composed of two transcriptionally distinct cell types

Supplementary Figure 7. STRING analyses for marker genes of ovary and testis

Supplementary Figure 8. Spatial transcriptomics suggests functional subsets of *F. hepatica* tubulins

Supplementary Figure 9. Spatial expression patterns of additional GSTs, ABC-B transporters, Ly6 proteins and PKCs

Supplementary Figure 10. Class and subfamily assignment of GSTs and ABC transporters by phylogenetic tree construction

Supplementary References

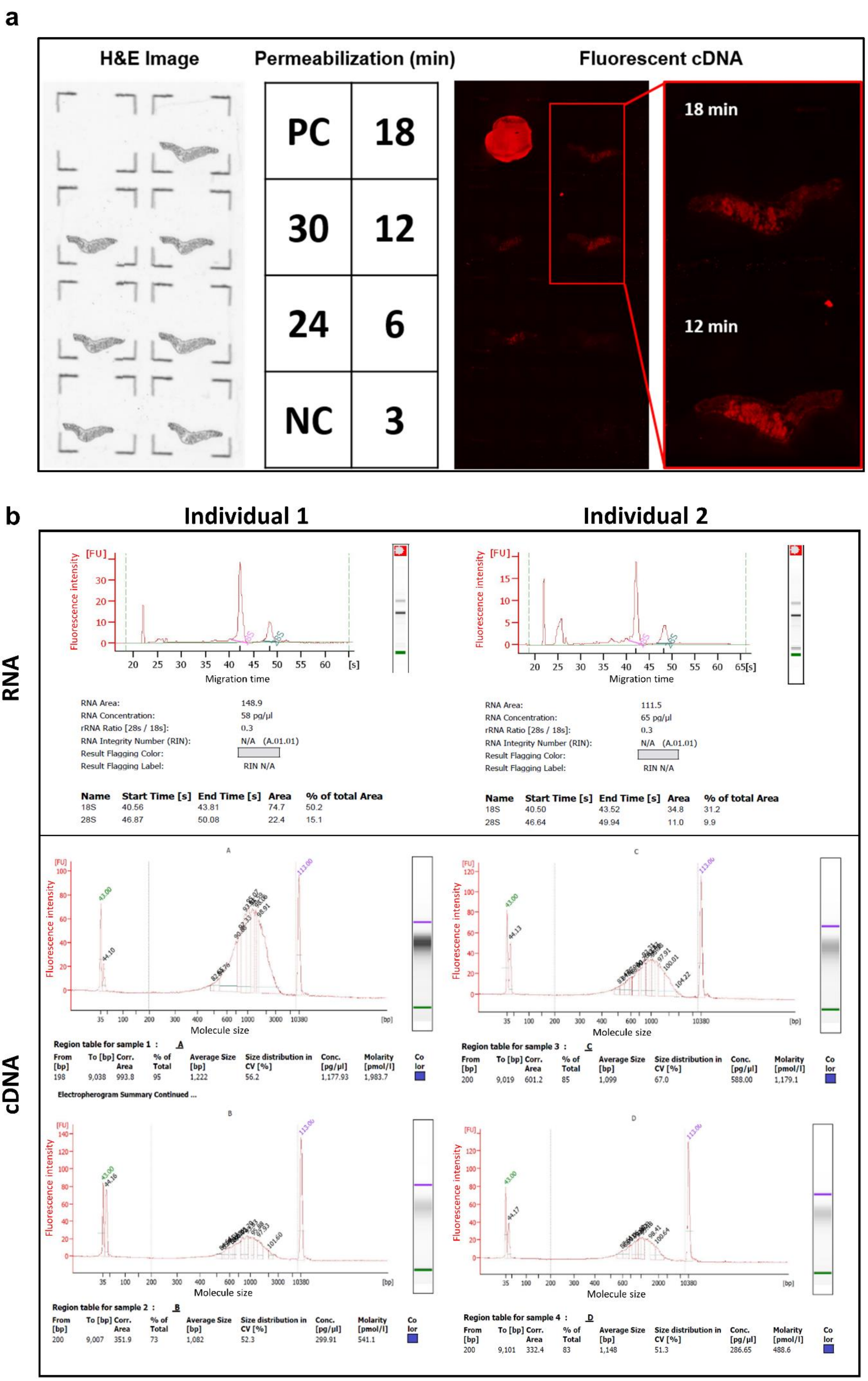
a
H&E Image
Permeabilization (min)
Fluorescent cDNA
PC
18
30
12
24
6
NC
3
18 min
12 min
b
Individual 1
Individual 2
RNA
cDNA
Fluorescence intensity
Migration time
RNA Area: 148.9
RNA Concentration: 58 pg/µl
rRNA Ratio [28s / 18s]: 0.3
RNA Integrity Number (RIN): N/A (A.01.01)
Result Flagging Color:
Result Flagging Label: RIN N/A
Name | Start Time [s] | End Time [s] | Area | % of total Area
18S | 40.56 | 43.81 | 74.7 | 50.2
28S | 46.87 | 50.08 | 22.4 | 15.1
RNA Area: 111.5
RNA Concentration: 65 pg/µl
rRNA Ratio [28s / 18s]: 0.3
RNA Integrity Number (RIN): N/A (A.01.01)
Result Flagging Color:
Result Flagging Label: RIN N/A
Name | Start Time [s] | End Time [s] | Area | % of total Area
18S | 40.50 | 43.52 | 34.8 | 31.2
28S | 46.64 | 49.94 | 11.0 | 9.9
Molecule size
Region table for sample 1 : A
From [bp] | To [bp] | Corr. Area | % of Total | Average Size [bp] | Size distribution in CV [%] | Conc. [pg/µl] | Molarity [pmol/l] | Color
198 | 9,038 | 993.8 | 95 | 1,222 | 56.2 | 1,177.93 | 1,983.7
Electropherogram Summary Continued ...
Region table for sample 3 : C
200 | 9,019 | 601.2 | 85 | 1,099 | 67.0 | 588.00 | 1,179.1
Region table for sample 2 : B
200 | 9,007 | 351.9 | 73 | 1,082 | 52.3 | 299.91 | 541.1
Region table for sample 4 : D
200 | 9,101 | 332.4 | 83 | 1,148 | 51.3 | 286.65 | 488.6

Supplementary Figure 1. 10x Visium preparation and tissue optimization

(a) The Visium Spatial Tissue Optimization Slide and Reagent Kit was used following manufacturer instructions (10x Genomics). The image on the left shows seven H&E-stained *Fasciola hepatica* cross-sections placed on a Visium Tissue Optimization slide. The frame on the upper left did not contain a tissue section, but a drop of *F. hepatica* RNA, which served as positive control. The table in the middle shows the permeabilization time each of these sections was treated with. Released mRNA was captured by oligonucleotides on the Visium Tissue Optimization slide and subsequently reverse transcribed into fluorescently labelled cDNA. The image on the right shows fluorescent cDNA produced from each of the sections and the positive control. 12 min permeabilization resulted in maximum fluorescence signal with the lowest signal diffusion (magnified view on the right). **(b)** Agilent Bioanalyzer profiles of RNA and cDNA derived from *F. hepatica* tissue sections. Upper panel: results of an RNA 6000 Pico Assay, one electropherogram for each specimen used in the spatial transcriptomics experiment. Lower Panel: results of a High Sensitivity DNA Assay, cDNA libraries derived from all four tissue sections (labelled A-D, as shown in Supplementary Fig. 2) used in the spatial transcriptomics experiment.

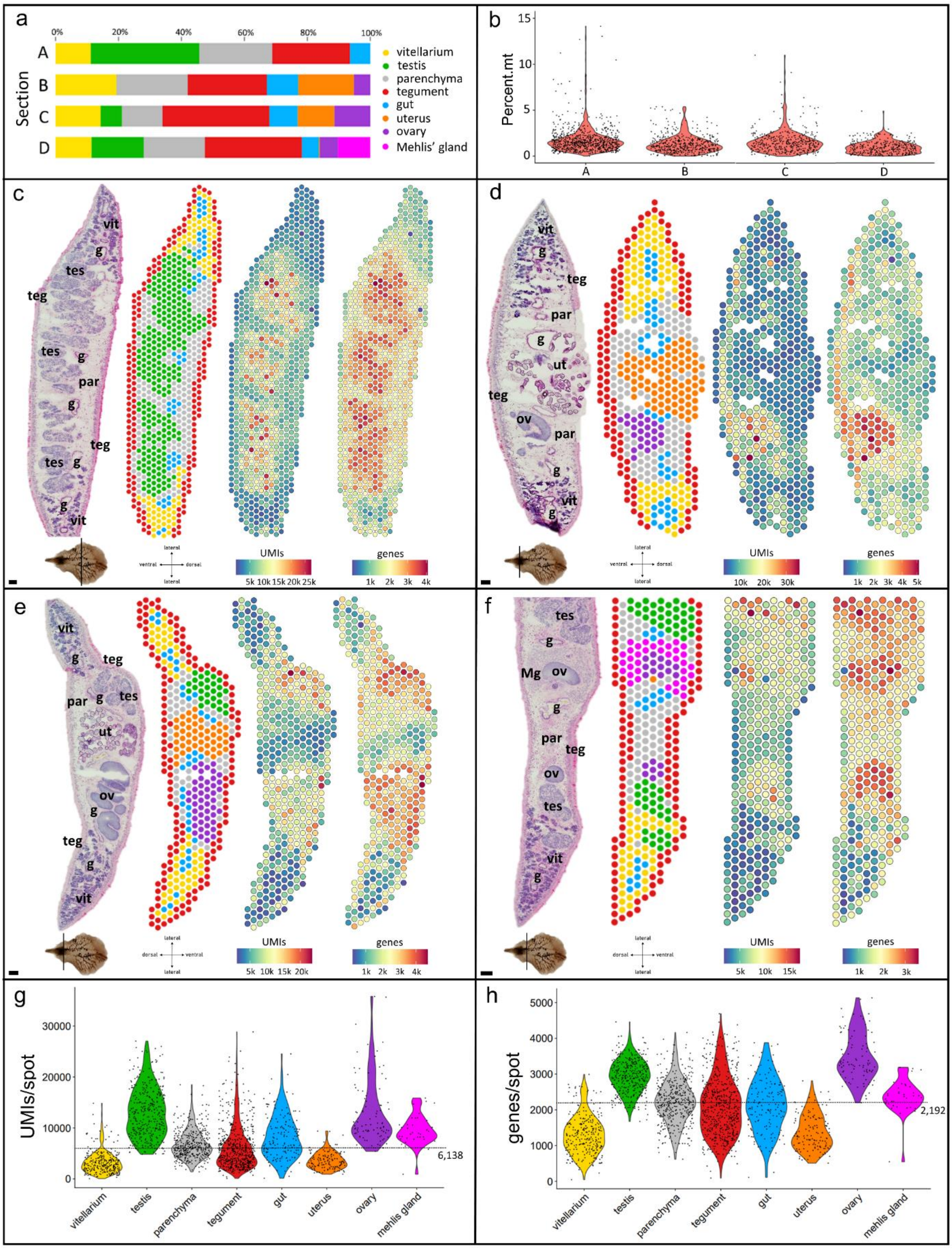

Supplementary Figure 2. Tissue composition and QC metrics of all four specimens in the liver fluke spatial transcriptomics dataset

(a) Tissue (cluster) composition of sections A-D. **(b)** Violin Plot showing the percentage of mitochondrial genes per spot split by section. **(c-f)** Left to right: H&E-stained tissue sections and corresponding spatial projections of 777, 481, 412 and 350 mRNA-binding spots covered by these tissue sections, respectively. Sectioning plane and orientation are indicated underneath. Scale = 100 µm, g: gut, Mg: Mehlis' gland, ov: ovary, par: parenchyma, teg: tegument, tes: testis, ut: uterus, vit: vitellarium. The first spatial projection shows the cluster assignment of all spots. Clusters are colored according to (a) and Figure 1. The second and third projection are displaying the distribution of UMI (Unique Molecular Identifier) and gene counts, respectively. The number of UMIs/ genes is encoded by color (blue – red = low – high). **(g,h)** Violin plot displaying UMI counts per spot (g) and gene counts per spot (f), split by cluster. The median UMI and gene count across all spots is indicated by the dashed line.

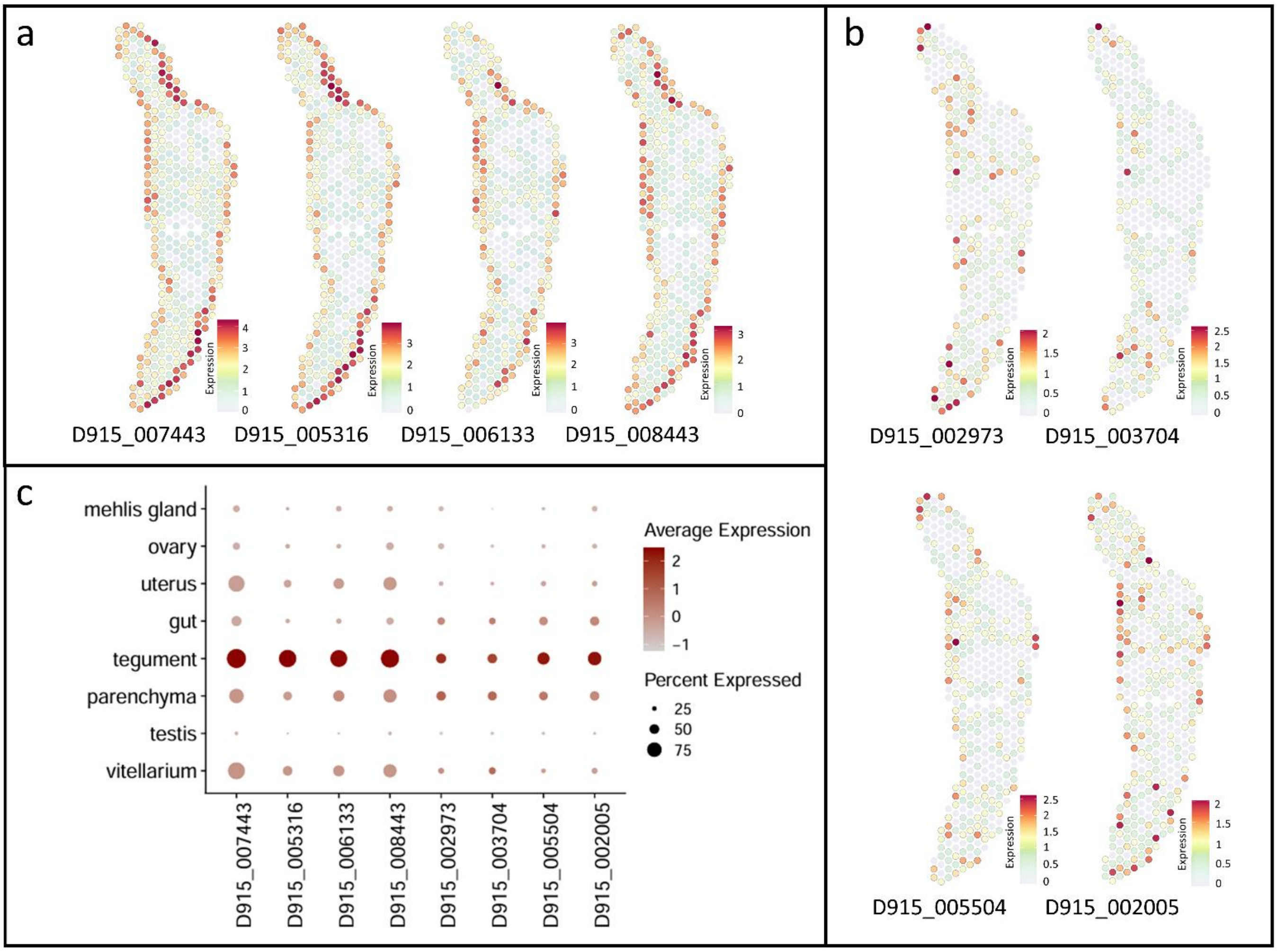

Supplementary Figure 3. Spatial expression of tegument and muscle markers

Tegumental and subtegumental muscle cells are both part of the *Fasciola hepatica* tegument cluster. Markers for tegument and muscle cells in *S. mansoni* were retrieved from scRNAseq data of Wendt et al. [S1]. The corresponding *F. hepatica* orthologues were retrieved from WormBase ParaSite. **(a)** Spatial projections showing expression profiles of four schistosome tegument-marker orthologues in *F. hepatica*: Actin (Smp_203130/D915_007443), Glucose transporter (Smp_105410/ D915_005316), Dynein (Smp_095520/D915_006133), Calcium-binding protein (Smp_267080/D915_008443). **(b)** Spatial projections showing expression profiles of four schistosome muscle-marker orthologs in *F. hepatica.* Tropomyosin 2 (Smp_031770/D915_002973), actin (Smp_307020, D915_003704), troponin (Smp_018250/D915_005504), paramyosin (Smp_085540/D915_002005). **(a,b)** Expression level encoded by color (grey = low, red = high). **(c)** DotPlot showing expression profiles of selected orthologs of schistosome tegument and muscle markers in *F. hepatica*. Dot color encodes the average expression level (= mean of UMI counts, normalized & scaled) across all spots within a cluster. Dot size encodes the percentage of spots within a cluster that have captured this transcript. Please note: While spatial plots (a,b) are shown for only one representative section, the DotPlot includes expression data from all four tissue sections in the data set. Source data for (c) are provided as Source Data file.

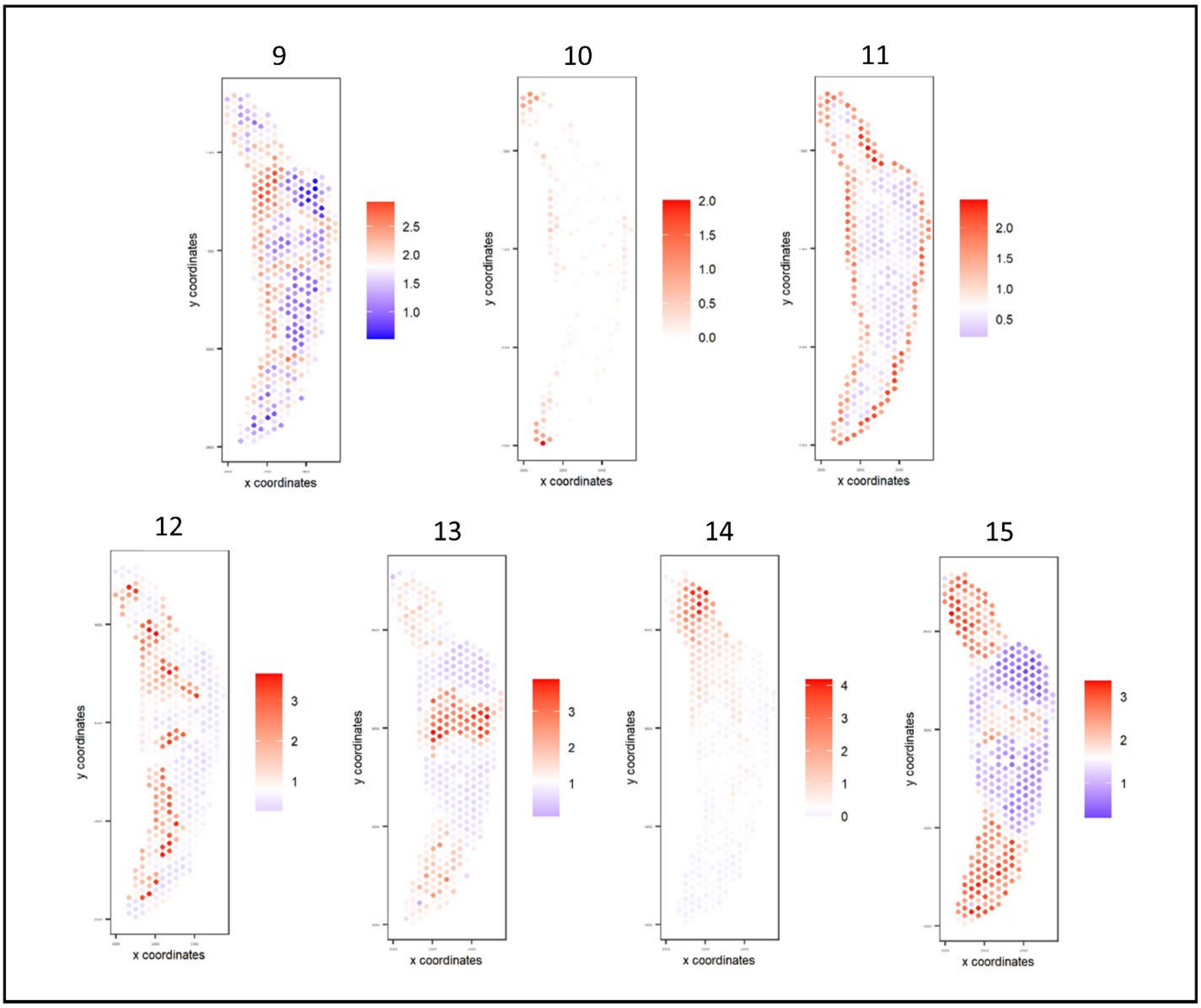

Supplementary Figure 4. Spatial expression of Giotto metagenes 9-15

Metagene visualizations for all spatial co-expression modules not shown in Figure 3.

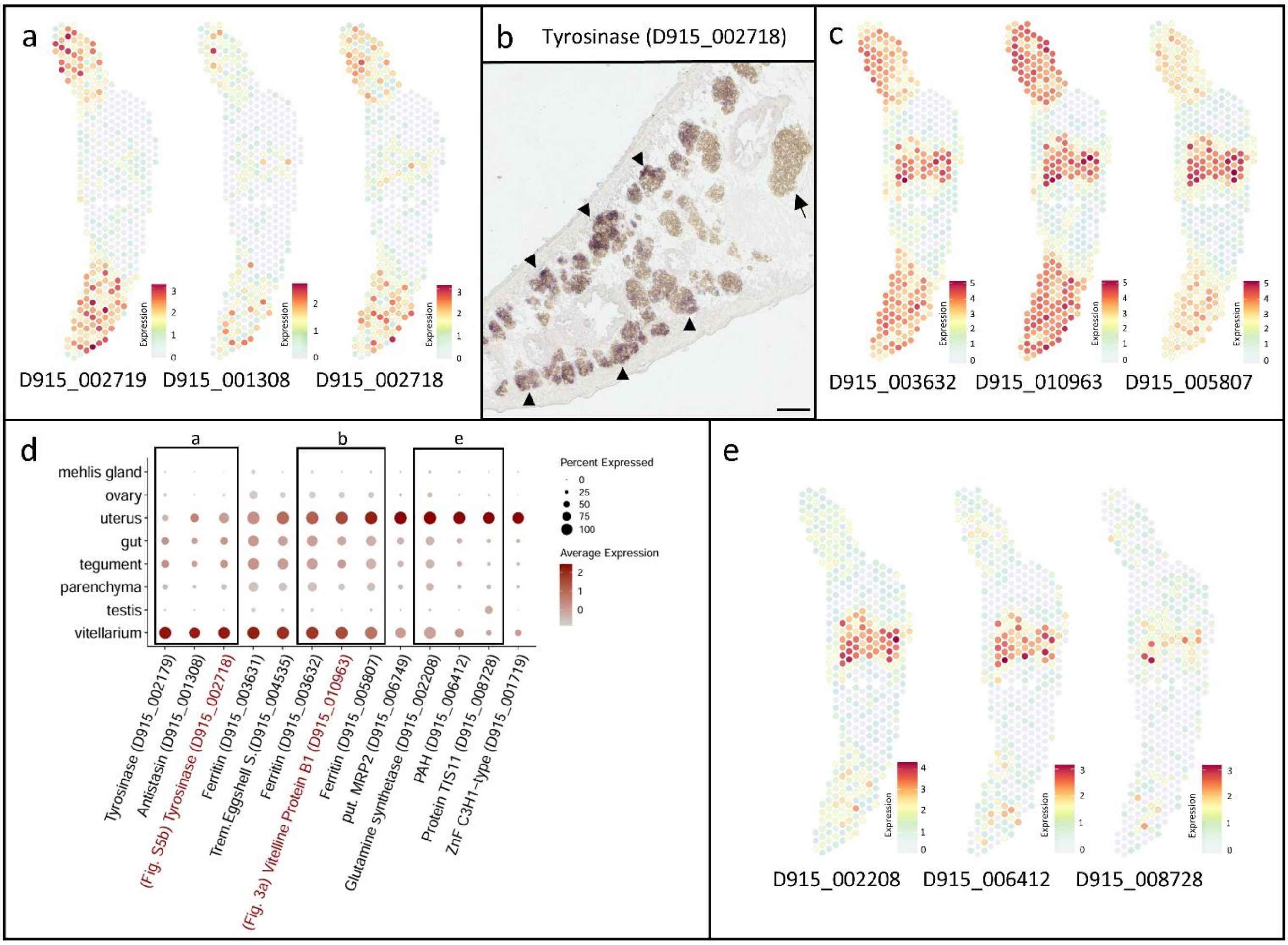

Supplementary Figure 5. The parasite's egg production apparatus: from vitellarium to uterus

(a-d) Trematode eggs are composite eggs, made up of an oocyte and about 30 vitelline cells surrounded by an eggshell [S2]. The lateral margins of adult liver flukes are filled with vitelline follicles, which provide vitelline cells that synthesize proteins and enzymes needed for eggshell formation [S3]. This includes eggshell proteins, tyrosinases and multiple iron-binding proteins such as ferritins and myoglobin. Spatial transcriptomics not only confirmed vitellarial expression, but could also shed light on the type of vitelline cell-stages that express particular genes. While tyrosinase 1 and 2 (D915_002197, D915_002718) were expressed exclusively within vitelline cells in the vitellarium (stages S1-S3), other markers, such as vitelline protein B1 (D915_010963), were also expressed in mature vitelline cells (stage S4) found within eggs in the uterus. (**a**) Spatial projections showing expression patterns of three genes with predominant expression in the vitellarium: tyrosinase 1 (D915_002197), Antistasin (D915_001308) and tyrosinase 2 (D915_002718). **(b)** Chromogenic ISH of the vitellarium-specific tyrosinase 2 (D915_002718). Black arrowheads indicate indigo dye deposition within vitelline follicles. No staining was observed within mature vitelline cells in the vitelline duct (black arrow). Scale = 100 µm. For number of experiments, see "Statistics and reproducibility" and Supplementary Data 9. **(c)** Spatial projections showing expression patterns of three genes with expression in both vitellarium and uterus: ferritin (D915_003632), vitelline protein B1 (D915_010963), and another ferritin (D915_005807). **(d, e)** The fluke's uterus is typically filled with eggs in different stages of eggshell formation, which was also the case for the specimens of the spatial transcriptomic analysis. Since each egg consists of dozens of S4 stage vitellocytes [S2], the uterus cluster shares several marker genes with the vitellarium cluster. Nevertheless, some genes that are predominantly expressed within the uterus could be identified that might be involved in early embryogenesis. This includes the glutamine synthetase D915_002208, the phenylalanine 4-monooxygenase (PAH) D915_006412 and the mRNA-binding protein TIS11 (D915_008728). **(d)** DotPlot showing expression profiles of selected tissue markers of the vitellarium and uterus cluster. Dot color encodes the average expression level (scaled) across all spots within a cluster. Dot size encodes the percentage of spots within a cluster that have captured this transcript. Please note: While spatial plots (a,c,e) are shown for only one representative section, the DotPlot includes expression data from all four tissue sections in the data set. Genes labelled in red were validated by (F)ISH. See Figure 4a for FISH of vitelline protein B1 (D915_010963). Boxes indicate the respective panel showing spatial projections of these genes. Source data are provided as Source Data file **(e)** Spatial projections showing expression patterns of three genes with predominant expression in the uterus: glutamine synthetase (D915_002208), phenylalanine 4-monooxygenase (D915_006412) and TIS11 (D915_008728). **(a,c,e)** Expression level encoded by color (grey = low, red = high).

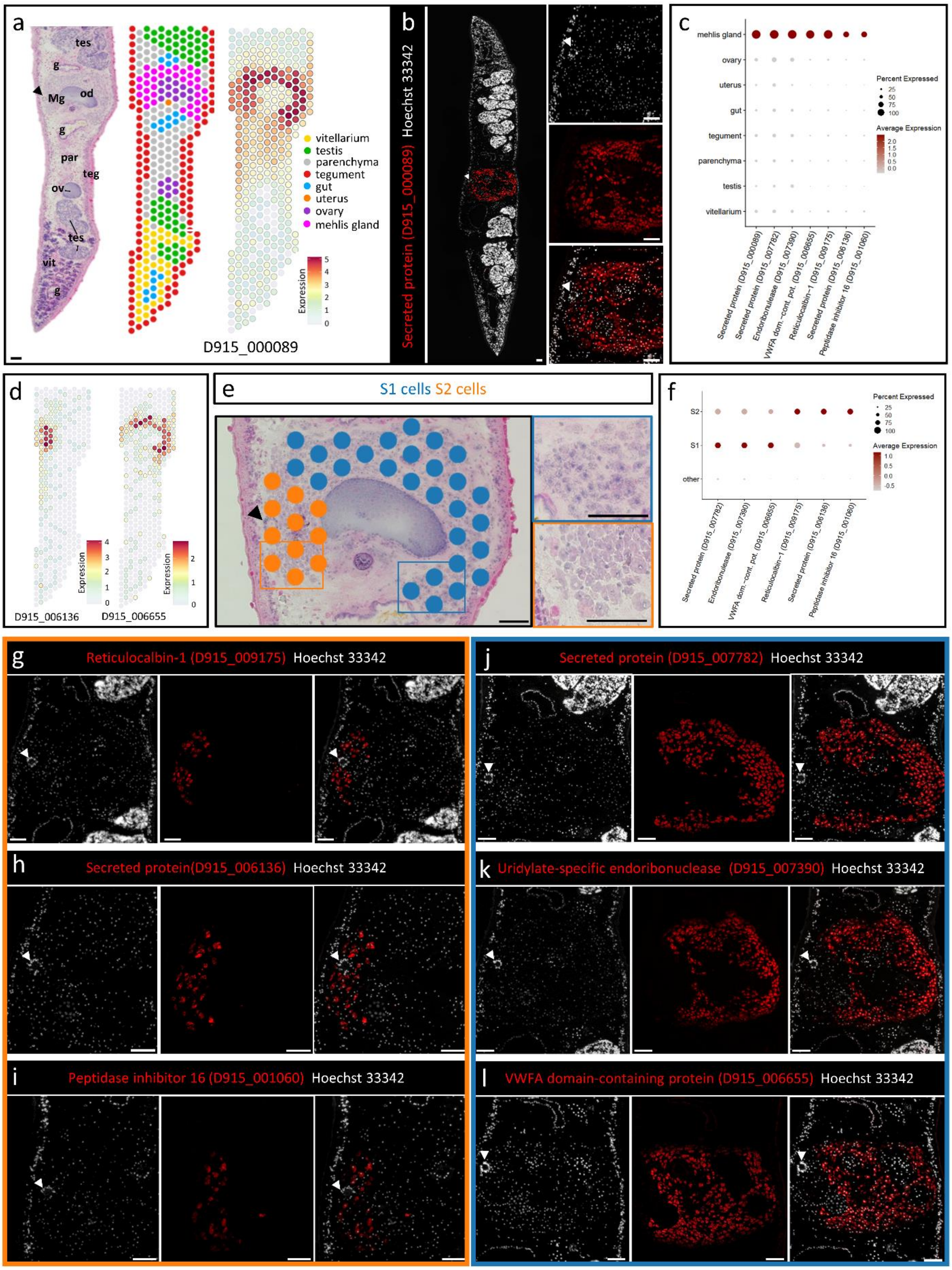

a
tes
g
Mg
od
g
par
teg
ov
tes
vit
g
vitellarium
testis
parenchyma
tegument
gut
uterus
ovary
mehlis gland
Expression
D915_000089
b
Secreted protein (D915_000089) Hoechst 33342
c
mehlis gland
ovary
uterus
gut
tegument
parenchyma
testis
vitellarium
Percent Expressed
Average Expression
Secreted protein (D915_000089)
Secreted protein (D915_007782)
Endoribonuclease (D915_007390)
VWFA dom.-cont. pot. (D915_006655)
Reticulocalbin-1 (D915_009175)
Secreted protein (D915_006136)
Peptidase inhibitor 16 (D915_001060)
d
Expression
D915_006136
D915_006655
e
S1 cells S2 cells
f
S2
S1
other
Percent Expressed
Average Expression
g
Reticulocalbin-1 (D915_009175) Hoechst 33342
h
Secreted protein(D915_006136) Hoechst 33342
i
Peptidase inhibitor 16 (D915_001060) Hoechst 33342
j
Secreted protein (D915_007782) Hoechst 33342
k
Uridylate-specific endoribonuclease (D915_007390) Hoechst 33342
l
VWFA domain-containing protein (D915_006655) Hoechst 33342

Supplementary Figure 6. The liver fluke's Mehlis' gland is composed of two transcriptionally distinct cell types

The Mehlis' gland or the so-called shell gland is part of the female reproductive apparatus and a unique feature of the reproductive system of parasitic flatworms [S4]. **(a)** Mehlis gland tissue was only included in one of our four specimens ("section D"). Left to right: H&E-stained tissue sections and corresponding spatial projections of 350 mRNA-binding spots covered by this tissue section. g: gut, Mg: Mehlis' gland, ov: ovary, par: parenchyma, teg: tegument, tes: testis, v: vitellarium. The first spatial projection shows the cluster assignment of all spots. Clusters are colored and labelled. The second spatial projection shows the spatial expression of the Mehlis' gland marker D915_000089. This secreted protein was tissue-wide expressed within the Mehlis' gland. Expression level encoded by color (grey = low, red = high). **(b)** D915_000089 expression was confirmed by fluorescent *in situ* hybridization (FISH). Overview (left) and detail (right). **(c)** DotPlot showing expression profiles of selected tissue markers of the Mehlis' gland cluster. Dot color encodes the average expression level (scaled) across all spots within a cluster. Dot size encodes the percentage of spots within a cluster that have captured this transcript. Please note: While spatial plots (a,d) are shown for only one representative section, the DotPlot includes expression data from all four tissue sections in the data set. Source data are provided as Source Data file. **(d)** Several marker genes of the Mehlis' gland cluster, e.g. another secreted protein (D915_006136) and a VWFA domain containing protein (D915_006655), showed a more localized expression within either the dorsal or the lateral and ventral part of the gland. Expression level encoded by color (grey = low, red = high). **(e)** These locations correspond to the arrangement of S1 (blue) and S2 cells (orange) within the Mehlis' gland of *F. hepatica* and have only been described morphologically so far [S5]. Having a closer look at the histology of the tissue section, it was also possible to detect distinct histological features described for those cell types: S1 cells are smaller and have a denser and basophilic cytoplasm compared to S2 cells which are larger and have a paler and more eosinophilic cytoplasm [S5].Therefore, we were able to manually select spots covering either S1 or S2 cells using Loupe Browser (10x Genomics). We then performed differential gene expression analysis in Seurat to compare the gene expression of both cell types. **(f)** DotPlot showing expression profiles of selected tissue markers of Mehlis' gland S1 & S2 cells. "Other" includes all remaining spots in the spatial dataset (all remaining clusters/tissues in all four sections). Dot color encodes the average expression level (scaled) across all spots within a cluster. Dot size encodes the percentage of spots within a cluster that have captured this transcript. Source data are provided as Source data file. For a complete list of cell type markers, see Supplementary Data 3. **(f-i)** FISH confirmed that S1 cells were characterized by the expression of Poly(U)-endoribonuclease (D915_007390), a von Willebrand factor A (VWFA) domain-containing protein (D915_006655), and a C3H1-type domain-containing protein (D915_007782). **(f, j-l)** For S2 cells, we identified reticulocalbin (D915_009175), peptidase inhibitor 16 (D915_001060) and another secreted protein (D915_006136) to be exclusively expressed within this cell type. **(a,b,e,g-l)** Scale = 100 µm, arrowhead marks Laurers' channel entering Mehlis' gland from dorsal. For numbers of ISH experiments performed for each gene, see "Statistics and reproducibility" and Supplementary Data 9.

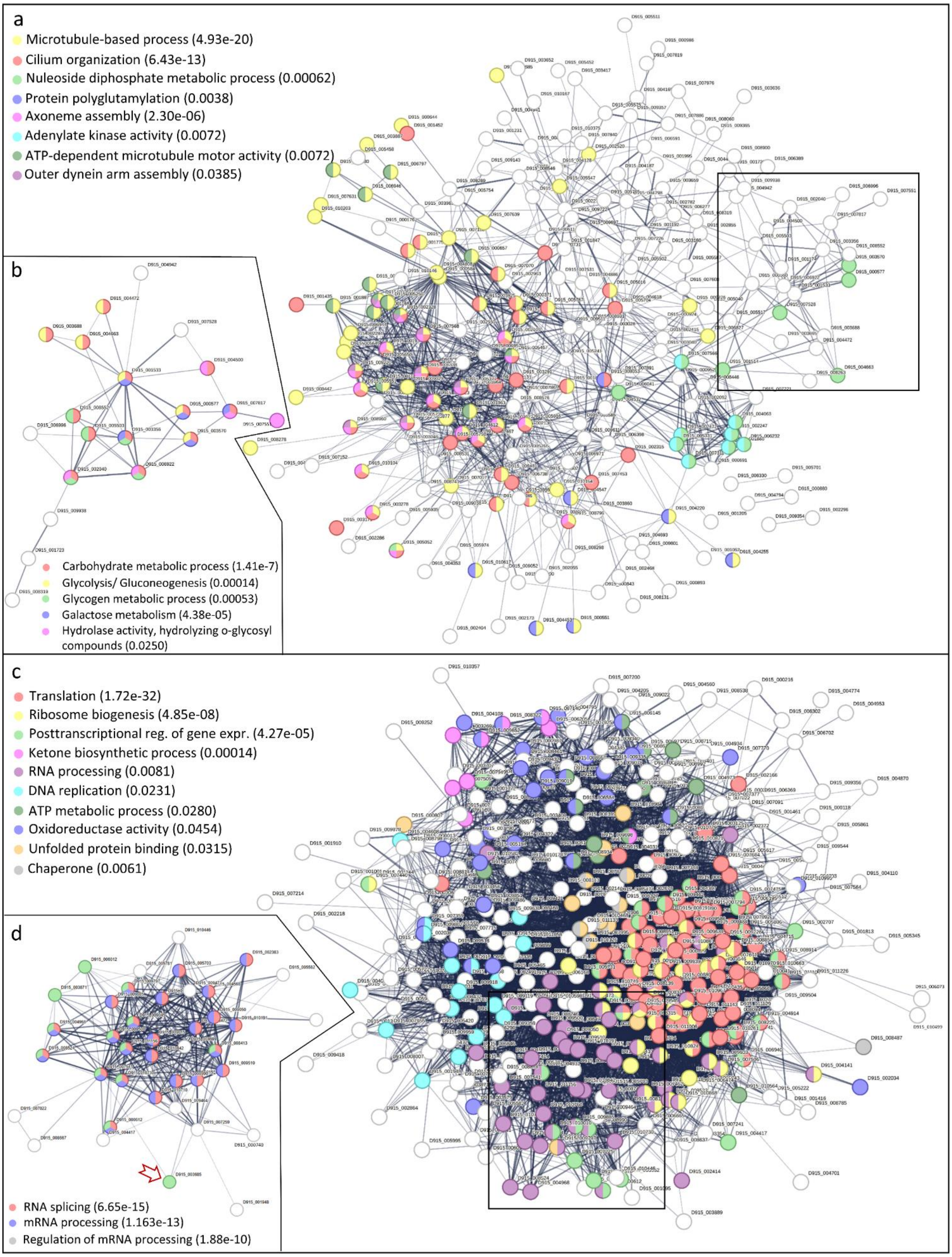

Supplementary Figure 7. STRING analyses for marker genes of ovary and testis

STRING analysis of marker genes (top 75%) for **(a)** testis and **(c)** ovary. Default settings were used to predict interactions with a minimum interaction (confidence) score of 0.4 (medium level of confidence). Lines (edges) are indicating interactions that were predicted based on the function of homologues. Disconnected nodes are not shown. Functional enrichments in the network were predicted by STRING. Selected terms are highlighted in color and labelled (false discovery rate in brackets). Subnetworks in **(b)** and **(c)** resulted from kmeans clustering in STRING (number of clusters = 10). Rectangles in (a) & (b) mark where the corresponding genes are located in the full network. **(d)** Arrow indicates Zinc finger CCCH domain-containing protein 3 (D915_003685).

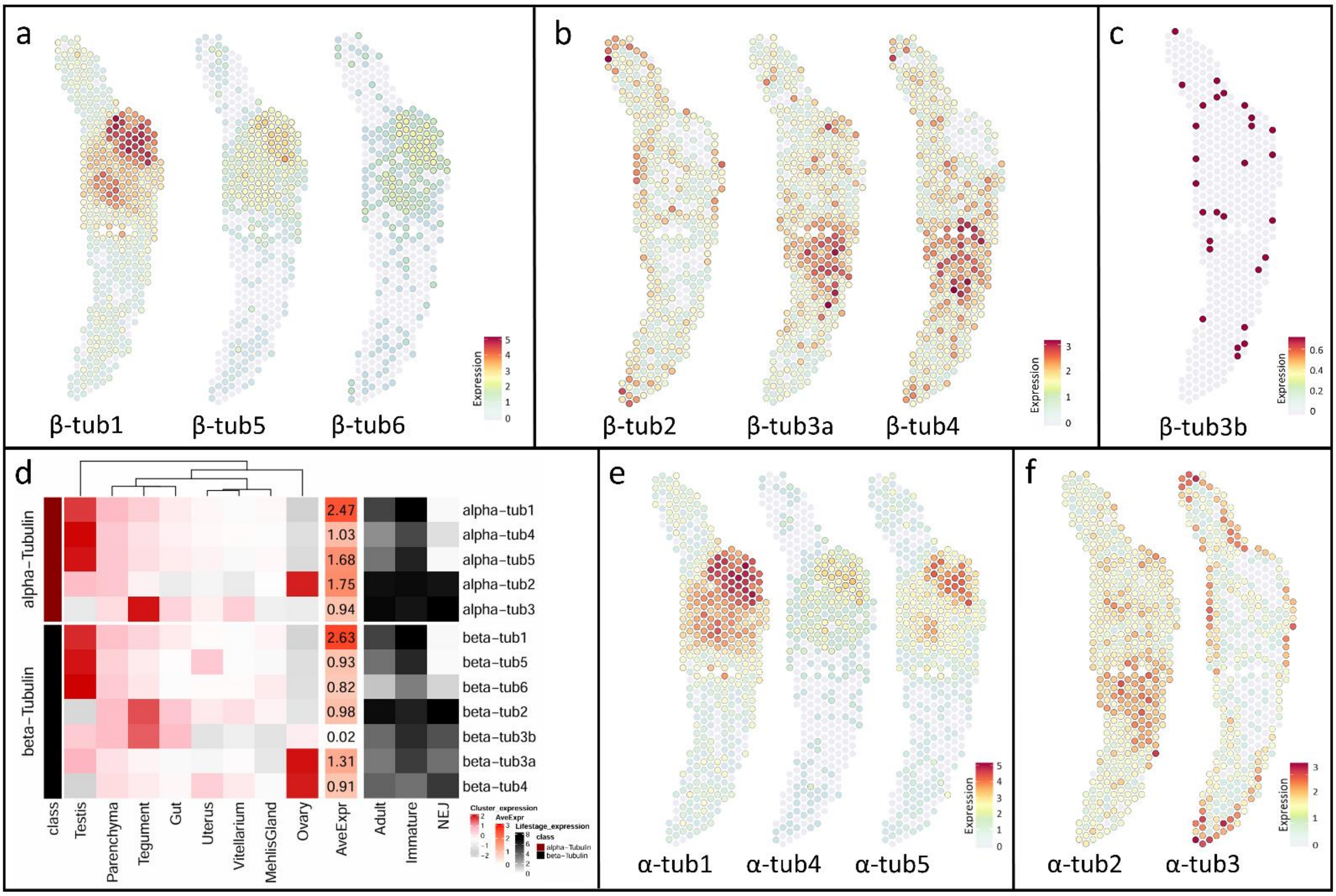

Supplementary Figure 8. Spatial transcriptomics suggests functional subsets of *F. hepatica* tubulins
Beta-tubulins are of research interest as they are molecular targets of triclabendazole, a benzimidazole and the drug of choice to treat fasciolosis [S6]. **(a-d)** All six *F. hepatica* β-tubulins (1-6) previously described in literature were found in the spatial transcriptome: β-tub1 (“beta-tub1”/D915_007398), β-tub2 (“beta-tub2”/D915_002311), β-tub3a (“beta-tub3.1”/D915_004911), β-tub3b (“beta-tub3”/ D915_002077), β-tub4 (“beta-tub4”/D915_001342), β-tub6 (“beta-tub6”/D915_008457). β-tubulin isoform 5 was found fragmented with D915_005076 and D915_003963 representing two halves of the complete β-tubulin isoform 5. Plots and heatmaps contain D915_003963. We were able to identify two major subsets of β-tubulins based on their spatial expression: β-tubulin 1, 5, and 6 were predominantly expressed in the testis (a,d), while isoforms 2, 3a, and 4 were expressed in a broader range of tissues (b-d). Isoforms 3a and 4 showed a peak expression in the flukes’ ovary (b,d). β-tubulin 2 in contrast was predominantly expressed in non-reproductive tissues, with highest expression in the tegument (b,d). The expression level of β-tubulin 3b (“beta-tub3”/ D915_002077) was relatively weak compared with the other isoforms and could not be confidently assigned to specific tissues. On average, its expression was highest in the tegument cluster (c,d). **(a-c)** Spatial projections showing expression patterns of *F. hepatica* β-tubulins. **(d)** Left: Heatmap showing the average expression of all α- and β-tubulins per cluster. Expression values were centered and scaled for each row (each gene) individually. Please note: While spatial plots (a-c & e,f) are shown for only one representative section, the heatmap includes expression data from all four tissue sections in the data set. Source data are provided as Source data file. Middle: Heatmap showing the average spot expression (AveExpr) of all α- and β-tubulins in the whole dataset (log1p normalized counts). Right: Heatmap showing tubulin life stage expression data from Cwiklinski et al [S7] (log1p normalized TPM values) for adult, immature and newly excysted juvenile *F. hepatica*. Mean values of 2-3 biological replicates. β-tubulin isoforms 2-4 showed an almost constitutive expression across all three life stages, implying a “housekeeping” role for general microtubule structure and function, whereas the remaining isoforms (β1, β5, β6) displayed a marked upregulation in immature and adult flukes, indicating a more specialized, but so far undetermined role [S7,S8]. Based on our spatial transcriptome, we can now conclude that this upregulation is probably due to a specific role of these isoforms in spermatogenesis. **(d-f)** The same holds true for α-tubulin isoforms 1, 4 & 5, which were also found testis-associated and upregulated in maturing parasites. **(e)** Spatial projections showing α-tubulin isoforms with predominant expression in the testis: α-tub1 (“alpha-tub1”/ D915_009242), α-tub4 (“alpha-tub4”/ D915_009559), α-tub5 (“alpha-tub5”/ “D915_005959). **(f)** Spatial projections of α-tub2 (“alpha-tub2”/ D915_005370) and α-tub3 (“alpha-tub3”/ D915_8616). **(a-c & e,f)** Expression level encoded by color (grey = low, red = high).

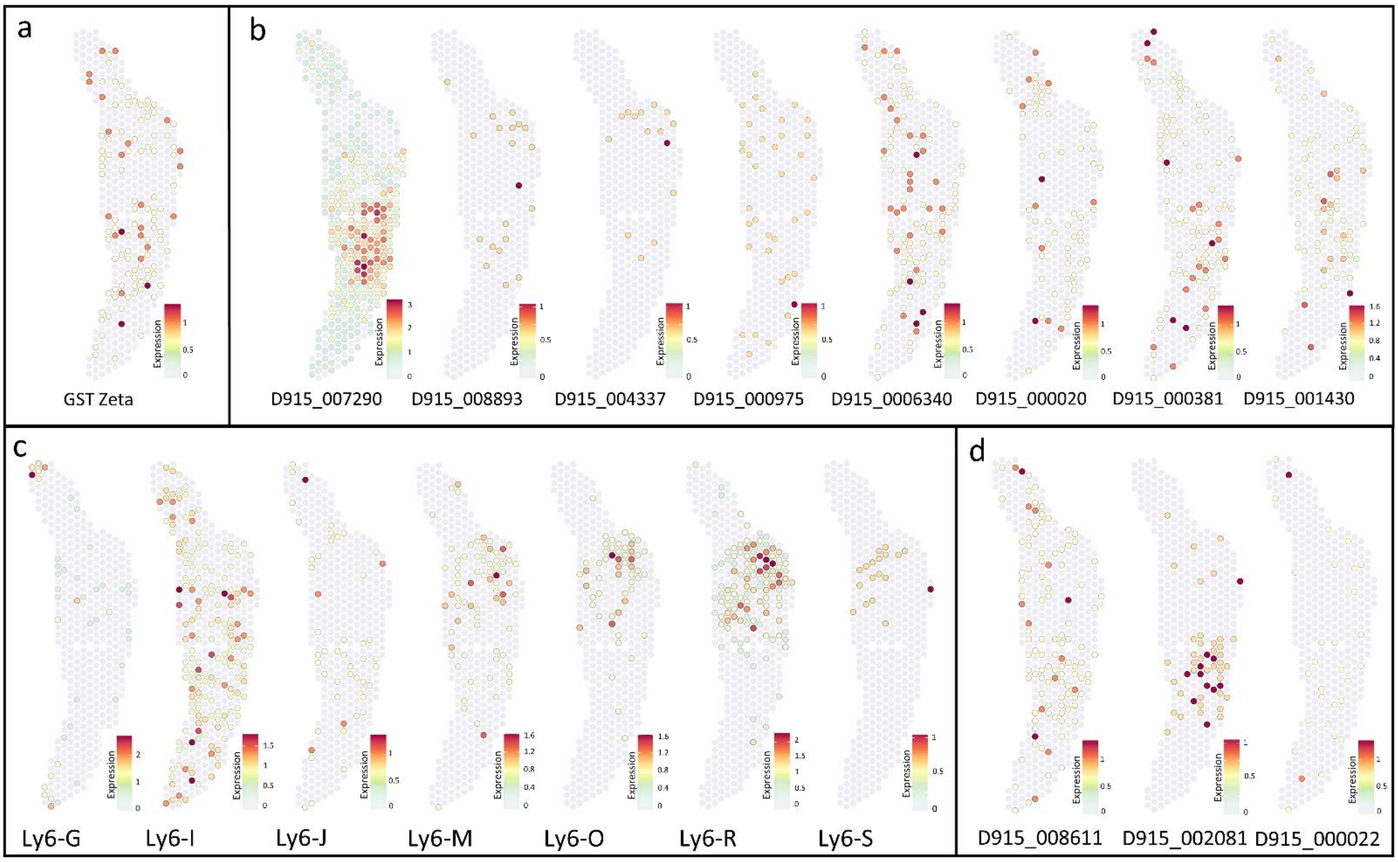

Supplementary Figure 9. Spatial expression patterns of additional GSTs, ABC-B transporters, Ly6 proteins and PKCs
(a) Spatial projection showing the expression pattern of zeta class GST (D915_006391), which was not shown in Figure 6a. **(b)** Spatial projections showing expression patterns of ABC transporters (subfamily B) not shown in Figure 6b. **(c)** Spatial projections showing expression patterns of *F. hepatica* Ly6 proteins not shown in Figure 6d: Ly6-G (D915_008997), Ly6-I (D915_002959), Ly6-J (D915_006710), Ly6-M (D915_008952), Ly6-O (D915_000989), Ly6-R (D915_000988), Ly6-S (D915_000991). **(d)** Spatial projections showing expression patterns of PKCs not shown in Figure 7d. **(a-d)** Expression level encoded by color (grey = low, red = high).

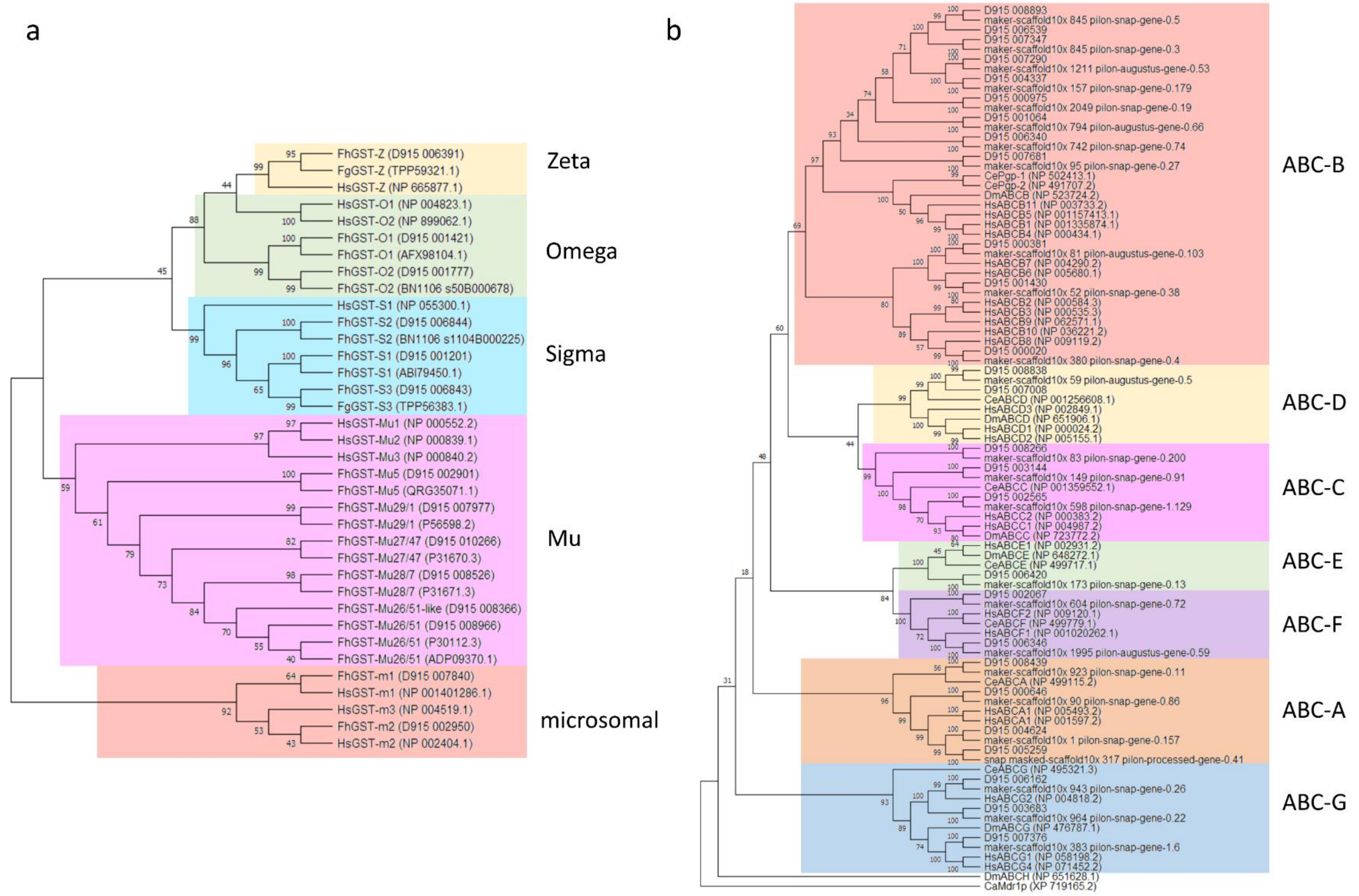

Supplementary Figure 10. Class and subfamily assignment of GSTs and ABC transporters by phylogenetic tree construction

(a) Phylogenetic analysis of *F. hepatica* (Fh) glutathione S-transferases (GSTs). *F. hepatica* GST amino acid sequences found in the genome of McNulty et al. [S9] (PRJNA179522,"D915"-IDs) were analyzed together with human (Hs) GST sequences as well as known *Fasciola* GST sequences. The latter were adopted from Stuart et al. [S10]. If no *F. hepatica* sequence was available, *F. gigantica* (Fg) sequences were used instead. Maximum likelihood analysis was performed in MegaX to 1000 bootstraps with JTT substitution. The tree was displayed as "Topology only". Microsomal GST sequences were used as outgroup. **(b)** Phylogenetic analysis of *F. hepatica* ATP-binding cassette (ABC) transporters. ABC transporter amino acid sequences of both *F. hepatica* genomes (PRJNA179522 with "D915"-IDs, PRJEB25283 with "marker-scaffold"-IDs) were analyzed together with human (Hs), *C. elegans* (Ce) and *D. melanogaster* (Dm) ABC sequences of all subfamilies (A-H). *Candida albicans* (Ca) Mdr1p was used as outgroup. ABC transporter gene IDs within PRJEB25283 were adopted from Beesley et al. [S11]. Maximum likelihood analysis was performed in MegaX to 1000 bootstraps with JTT substitution. The tree was displayed as "Topology only".

Supplementary References

S1 Wendt, G. *et al.* A single-cell RNA-seq atlas of *Schistosoma mansoni* identifies a key regulator of blood feeding. *Science* **369,** 1644–1649; 10.1126/science.abb7709 (2020).

S2 Smyth, J. D. & Halton, D. W. The biology of the egg. In *The physiology of trematodes,* edited by J. D. Smyth & D. W. Halton. 2nd ed. (Cambridge University Press, Cambridge, 1983), pp. 86–114.

S3 Hanna, R. E. B., Fairweather, I. & Robinson, M. W. 4 The reproductive system of *Fasciola hepatica*. 4.6.1 Egg formation. In *Fasciolosis,* edited by J. P. Dalton. 2nd ed. (CAB International, Wallingford, 2021), pp. 133–137.

S4 Smyth, J. D. & Clegg, J. A. Egg-shell formation in trematodes and cestodes. *Exp Parasitol* **8,** 286–323; 10.1016/0014-4894(59)90027-X (1959).

S5 Hanna, R. *Fasciola hepatica*: Histology of the reproductive organs and differential effects of triclabendazole on drug-sensitive and drug-resistant fluke isolates and on flukes from selected field cases. *Pathogens* **4,** 431–456; 10.3390/pathogens4030431 (2015).

S6 Fairweather, I., Brennan, G. P., Hanna, R. E. B., Robinson, M. W. & Skuce, P. J. Drug resistance in liver flukes. *Int J Parasitol Drugs Drug Resist* **12,** 39–59; 10.1016/j.ijpddr.2019.11.003 (2020).

S7 Cwiklinski, K. *et al.* The *Fasciola hepatica* genome: gene duplication and polymorphism reveals adaptation to the host environment and the capacity for rapid evolution. *Genome Biol* **16**; 10.1186/s13059-015-0632-2 (2015).

S8 Cwiklinski, K. & Dalton, J. P. Omics tools enabling vaccine discovery against fasciolosis. *Trends Parasitol* **38,** 1068–1079; 10.1016/j.pt.2022.09.009 (2022).

S9 McNulty, S. N. et al. Genomes of *Fasciola hepatica* from the Americas reveal colonization with Neorickettsia endobacteria related to the agents of Potomac horse and human Sennetsu fevers. *PLOS Genet* **13**; 10.1371/journal.pgen.1006537 (2017).

S10 Stuart, R. B. *et al.* The soluble glutathione transferase superfamily: role of Mu class in triclabendazole sulphoxide challenge in *Fasciola hepatica*. *Parasitol Res* **120,** 979–991; 10.1007/s00436-021-07055-5 (2021).

S11 Beesley, N. J. *et al.* A major locus confers triclabendazole resistance in *Fasciola hepatica* and shows dominant inheritance. *PLoS Pathog* **19,** e1011081; 10.1371/journal.ppat.1011081 (2023).

S12 Robb, E. *et al.* Transcriptomic analysis supports a role for the nervous system in regulating growth and development of *Fasciola hepatica* juveniles. *PLOS Negl Trop Dis* **16,** e0010854; 10.1371/journal.pntd.0010854 (2022).

S13 Davey, S. D. *et al. In silico* characterisation of the complete Ly6 protein family in *Fasciola gigantica* supported through transcriptomics of the newly-excysted juveniles. *Mol Omics* **18,** 45–56; 10.1039/D1MO00254F (2022).

S14 Shi, Y. *et al.* First insight into CD59-like molecules of adult *Fasciola hepatica*. *Exp Parasitol* **144**, 57–64; 10.1016/j.exppara.2014.06.012 (2014).

S15 Rice-Ficht, A. C., Dusek, K. A., Kochevar, G. J. & Waite, J. H. Eggshell precursor proteins of *Fasciola hepatica*, I. Structure and expression of vitelline protein B. *Mol Biochem Parasitol* **54,** 129–141; 10.1016/0166-6851(92)90106-T (1992).
