# Supplementary material for: Spatial transcriptomics of a parasitic flatworm provides a molecular map of drug targets and drug resistance genes
Source: Nat Commun. 2024 Oct 16;15:8918. doi: 10.1038/s41467-024-53215-3 (PMC11484910; doi:10.1038/s41467-024-53215-3)
Supplement: Supplementary file 3 — Description of additional supplementary files [file 41467_2024_53215_MOESM3_ESM.pdf]

Description of Additional Supplementary Files

Supplementary Data 1. List of samples used for spatial transcriptomics and corresponding metrics.

Supplementary Data 2. List of marker genes per cluster: The FindAllMarkers() function embedded in Seurat was used to identify markers for each of the clusters in the spatial transcriptomics dataset by “ROC” test.

Supplementary Data 3. Differential gene expression of Mehlis' gland S1 and S2 cells: The FindMarkers() function embedded in Seurat was used to identify upregulated genes in S1 vs S2 cells and vice versa by performing a two-sided Wilcoxon Rank Sum test.

Supplementary Data 4. Giotto metagene composition.

Supplementary Data 5. List of cell cycle- and stem cell-associated genes in Fasciola hepatica.

Supplementary Data 6. Summary of all FhLy6 proteins.

Supplementary Data 7. List of predicted targets and drugs in the Fasciola hepatica tegument and gut

Supplementary Data 8. Reagents & chemicals used within the Visium Spatial Gene Expression workflow in deviation from manufacturer's instructions.

Supplementary Data 9. Riboprobes for in situ hybridization: Cloning information for all markers used for ISH validation and number of independent in situ hybridization experiments performed per marker gene.

Supplementary Movie 1. Adult Fasciola hepatica depicts normal motility (score 3) after 72 h treatment with DMSO as control.

Supplementary Movie 2. Adult Fasciola hepatica with a motility score of 0 (dead) after 72 h treatment with 50 µM ruboxistaurin.
