# Supplementary material for: Spatial transcriptomics of a parasitic flatworm provides a molecular map of drug targets and drug resistance genes
Source: Nat Commun. 2024 Oct 16;15:8918. doi: 10.1038/s41467-024-53215-3 (PMC11484910; doi:10.1038/s41467-024-53215-3)
Supplement: Supplementary file 7 — Reporting Summary [file 41467_2024_53215_MOESM7_ESM.pdf]

nature portfolio

Corresponding author(s): Simone Haeberlein

Last updated by author(s): Sep 12, 2024

Reporting Summary

Nature Portfolio wishes to improve the reproducibility of the work that we publish. This form provides structure for consistency and transparency in reporting. For further information on Nature Portfolio policies, see our Editorial Policies and the Editorial Policy Checklist.

Statistics

For all statistical analyses, confirm that the following items are present in the figure legend, table legend, main text, or Methods section.

n/a	Confirmed	
☐	☒	The exact sample size (*n*) for each experimental group/condition, given as a discrete number and unit of measurement
☐	☒	A statement on whether measurements were taken from distinct samples or whether the same sample was measured repeatedly
☐	☒	The statistical test(s) used AND whether they are one- or two-sided<br>*Only common tests should be described solely by name; describe more complex techniques in the Methods section.*
☒	☐	A description of all covariates tested
☒	☐	A description of any assumptions or corrections, such as tests of normality and adjustment for multiple comparisons
☐	☒	A full description of the statistical parameters including central tendency (e.g. means) or other basic estimates (e.g. regression coefficient) AND variation (e.g. standard deviation) or associated estimates of uncertainty (e.g. confidence intervals)
☐	☒	For null hypothesis testing, the test statistic (e.g. *F*, *t*, *r*) with confidence intervals, effect sizes, degrees of freedom and *P* value noted<br>*Give P values as exact values whenever suitable.*
☒	☐	For Bayesian analysis, information on the choice of priors and Markov chain Monte Carlo settings
☒	☐	For hierarchical and complex designs, identification of the appropriate level for tests and full reporting of outcomes
☒	☐	Estimates of effect sizes (e.g. Cohen's *d*, Pearson's *r*), indicating how they were calculated

Our web collection on statistics for biologists contains articles on many of the points above.

Software and code

Policy information about availability of computer code

Data collection

RNA & cDNA QC
-Agilent 2100 Bioanalyzer

Sequencing of spatially barcoded cDNA libraries:
- Illumina Novaseq 6000

H&E Imaging data preprocessing (Tissue detection and fiducial frame alignement, manual)
- Loupe Browser (v6.0.0) 10xGenomics https://www.10xgenomics.com/support/software/loupe-browser

Mapping of reads to the reference transcriptome and spot barcodes
- Space Ranger (v1.3.1) 10xGenomics https://www.10xgenomics.com/support/software/space-ranger

Microscopy:
- H&E-stained tissue sections for 10x Visium: LAS X (Leica)
- (F)ISH: cellSens Dimension (Olympus) or LAS X (Leica)

Data analysis

Clustering, Marker gene analysis and vizualization:
--> The analysis was mainly based on the vignette "Analysis, visualization, and integration of spatial datasets with Seurat" (https://satijalab.org/seurat/articles/spatial_vignette.html#x-visium). For batch correction using Harmony, as well as export & re-import from Seurat to Loupe Browser, we used the Analysis Guide "Correcting Batch Effects in Visium Data" on the 10xGenomics webpage as a reference (https://www.10xgenomics.com/resources/analysis-guides/correcting-batch-effects-in-visium-data). The code provided by Soria et. al (https://

doi.org/10.5281/zenodo.4081608) served as a template for GO term enrichment analysis using topGO.

The combined code used for data analysis in this study has been deposited at Zenodo (https://doi.org/10.5281/zenodo.10245261) and is publicly available.

Software/ Packages used:
- Seurat (v4.3.0.) Hao et al. (2021), 10.1016/j.cell.2021.04.048 , https://satijalab.org/seurat/
- Harmony (v0.1.1) Korsunsky et al. (2019), 10.1038/s41592-019-0619-0, https://github.com/immunogenomics/harmony
- ComplexHeatmap (v2.10.0) Gu et al. (2016), 10.1093/bioinformatics/btw313, Bioconductor: 10.18129/B9.bioc.ComplexHeatmap

Spatial co-expression analysis
-Giotto (v4.0.5) Dries et al. 2021, https://doi.org/10.1186/s13059-021-02286-2, https://drieslab.github.io/Giotto_website/articles/installation.html

GO term anotation and enrichment analysis
- InterProScan (v5.60.92.19) Jones et al. (2014), 10.1093/bioinformatics/btu031, https://ftp.ebi.ac.uk/pub/software/unix/iprscan/5/5.60-92.0/interproscan-5.60-92.0-64-bit.tar.gz
- topGO (v2.46.0) Alexa and Rahnenfuhrer (2021), 10.18129/B9.BIOC.TOPGO, https://bioconductor.org/packages/release/bioc/html/topGO.html

Prediction of Protein-Protein interactions
- STRING (v11.5) Szklarczyk et al. (2019), 10.1093/nar/gky1131 , https://version-11-5.string-db.org/organism/STRG0085JJO

Protein domain detection:
- SMART Letunic et al. (2021), http://smart.embl-heidelberg.de/

Multiple Alignement and phylogenetic tree construction:
- Clustal Omega (v1.2.4) Madeira et al. (2022), 10.1093/nar/gkac240, https://www.ebi.ac.uk/Tools/msa/clustalo/
- MEGA X (v10.2.4) Kumar et al. (2018), 10.1093/molbev/msy096 , https://www.megasoftware.net/downloads/dload_win_gui

Drug and target database:
ChEMBL (v34) Zdrazil et al. (2024), 10.1093/nar/gkad1004 , 10.6019/CHEMBL.database.34
accessed via: DB Browser for SQLite (v3.12.2), https://sqlitebrowser.org/dl/

BLAST
- BLAST+ (v2.13.0), NCBI, Camacho et al. (2009), 10.1186/1471-2105-10-421, https://ftp.ncbi.nlm.nih.gov/blast/executables/blast+/2.13.0/

Image processing & Figure arrangement:
- Fiji (ImageJ, v1.54f) Schindelin et al. (2012), 10.1038/nmeth.2019, https://fiji.sc/
- Microsoft Office PowerPoint 2016

Scoring data: data presentation and statistics:
- GraphPad Prism (v8)

Online platform for data visualization:
- Cirrocumulus Li et al. (2020), 10.1038/s41592-020-0905-x, https://github.com/lilab-bcb/cirrocumulus

For manuscripts utilizing custom algorithms or software that are central to the research but not yet described in published literature, software must be made available to editors and reviewers. We strongly encourage code deposition in a community repository (e.g. GitHub). See the Nature Portfolio guidelines for submitting code & software for further information.

Data

Policy information about availability of data

All manuscripts must include a data availability statement. This statement should provide the following information, where applicable:

- Accession codes, unique identifiers, or web links for publicly available datasets
- A description of any restrictions on data availability
- For clinical datasets or third party data, please ensure that the statement adheres to our policy

All raw sequence data was deposited in the SRA under accession number PRJNA1047549 (https://www.ncbi.nlm.nih.gov/bioproject/PRJNA1047549) and is publicly available.

Filtered feature barcode matrices, slide images and manually corrected barcode-cluster assignments have been deposited at Zenodo (https://doi.org/10.5281/zenodo.10245261) and is publicly available

Analyzed data can be visualized and explored in Cirrocumulus using the following link: https://www.uni-giessen.de/haeberlein-lab/en/info. Alternatively, a .cloupe file can be downloaded from Zenodo (https://doi.org/10.5281/zenodo.10245261) to explore the data using LoupeBrowser (10x Genomics).

The F. hepatica proteome (PRJNA179522), uploaded to the STRING database, is accessible via the organism identifier STRG0085JJO [https://version-11-5.string-db.org/organism/STRG0085JJO]

Research involving human participants, their data, or biological material

Policy information about studies with human participants or human data. See also policy information about sex, gender (identity/presentation), and sexual orientation and race, ethnicity and racism.

Reporting on sex and gender	Does not apply
Reporting on race, ethnicity, or other socially relevant groupings	Does not apply
Population characteristics	Does not apply
Recruitment	Does not apply
Ethics oversight	Does not apply

Note that full information on the approval of the study protocol must also be provided in the manuscript.

Field-specific reporting

Please select the one below that is the best fit for your research. If you are not sure, read the appropriate sections before making your selection.

☒ Life sciences ☐ Behavioural & social sciences ☐ Ecological, evolutionary & environmental sciences

For a reference copy of the document with all sections, see nature.com/documents/nr-reporting-summary-flat.pdf

Life sciences study design

All studies must disclose on these points even when the disclosure is negative.

Sample size	No sample size calculation was performed. For spatial transcriptomics, we analysed the spatial transcriptome of n=4 tissue sections derived from two individual parasites (2x2).<br><br>For in situ hybridization, we used n=2-5 tissue sections of different body regions of a single individuum in each independent experiment. Replicates were performed on tissue sections deriving from distinct parasite individuals.<br><br>For inhibitor testings, two worms were used per group and independent experiment. In total, we used n=4-6 individual worms per condition.
Data exclusions	For spatial transcricptomics, only spots covered by parasite tissues (selected manually) were included in the analysis. Data derived from empty spots was excluded. No filtering was applied during the following analysis.<br><br>No data was excluded from the analysis of inhibitor treatments.
Replication	The spatial transcriptomics workflow was performed once. Tissue sections from different parasites (collected from different animals) were considered biological replicates.<br>ISH experiments were performed independently 1-3x, using sections from different parasite individuals in each experiment (for detailed numbers of independent experiments per gene, see Supplementary Data 9.<br>Inhibitor experiments were independently repeated 2-3x (Technical replicates). Different parasite individuals were considered biological replicates.<br>Replications were successful in delivering reproducible results.
Randomization	For spatial transcriptomics, each replicate (individual parasite) was randomly selected from a pool of individuals collected from the host animal. Tissue regions were sampled purpously from different body regions to include distinct organs of interest (see Methods for details)<br>For in situ hybridization, parasite individuals and tissue sections were randomly allocated to different stainings.<br>For inhibitor testing, worms harvested from host animals were randomly allocated to either the inhibitor or the control group.
Blinding	For inhibitor treatments, investigators were not blinded to allocation during experiments and outcome assessment.

Reporting for specific materials, systems and methods

We require information from authors about some types of materials, experimental systems and methods used in many studies. Here, indicate whether each material, system or method listed is relevant to your study. If you are not sure if a list item applies to your research, read the appropriate section before selecting a response.

Materials & experimental systems

n/a	Involved in the study
☐	☒ Antibodies
☒	☐ Eukaryotic cell lines
☒	☐ Palaeontology and archaeology
☐	☒ Animals and other organisms
☒	☐ Clinical data
☒	☐ Dual use research of concern
☒	☐ Plants

Methods

n/a	Involved in the study
☒	☐ ChIP-seq
☒	☐ Flow cytometry
☒	☐ MRI-based neuroimaging

Antibodies

Antibodies used

Anti-Digoxigenin-POD, Fab fragments, Roche, 11207733910
Anti-Digoxigenin-AP, Fab fragments, Roche, 11093274910

Validation

General description from manufactures webpage (same for both antibodies):
Digoxigenin is a hapten, useful in labeling and detection of nucleic acids. This product contains Fab fragments from polyclonal anti-digoxigenin antibodies, conjugated to alkaline phosphatase. Anti-Digoxigenin-AP, Fab fragments are useful for the detection of digoxigenin-labeled compounds.
Specificity: The polyclonal antibody from sheep is specific to digoxigenin and digoxin and shows no cross-reactivity with other steroids, such as human estrogens and androgens.
Analysis note (Anti Digoxigenin-AP):
(...) Nonspecific binding to RNA is not expected
(https://www.sigmaaldrich.com/DE/en/product/roche/11207733910
https://www.sigmaaldrich.com/DE/en/product/roche/11093274910)

Animals and other research organisms

Policy information about studies involving animals; ARRIVE guidelines recommended for reporting animal research, and Sex and Gender in Research

Laboratory animals

Male Wistar rats RjHan:WI (Rattus norvegicus) (Janvier, France) were used as model hosts for F. hepatica infection. Rats were orally infected with 25 metacercariae of an Italian parasite strain (purchased from Ridgeway Research, UK) at an age of 4-6 weeks and sacrificed 12-14 weeks later to collect adult parasites.

Wild animals

The study did not involve wild animals.

Reporting on sex

For husbandry reasons, all host animals were male rats.
Most trematodes (inkl. F. hepatica) are hermaphrodites with both male and female reproductive organs. There are no individuals with separate sexes.

Field-collected samples

The study did not involve samples collected from the field.

Ethics oversight

Animal experiments were performed in accordance with Directive 2010/63/EU on the protection of animals used for scientific purposes and the German Animal Welfare Act. The experiments were approved by the Regional Council (Regierungspraesidium) Giessen (V54-19c20 15 h 02 GI 18/10 Nr. A16/2018).

Note that full information on the approval of the study protocol must also be provided in the manuscript.

Plants

Seed stocks

Does not apply

Novel plant genotypes

Does not apply

Authentication

Does not apply
